# Protection of dopamine neurons by CDNF and neurturin variant N4 against MPP^+^ in dissociated cultures from rat mesencephalon

**DOI:** 10.1371/journal.pone.0245663

**Published:** 2021-02-03

**Authors:** Juliann D. Jaumotte, Mart Saarma, Michael J. Zigmond

**Affiliations:** 1 Department of Neurology, University of Pittsburgh, Pittsburgh, PA, United States of America; 2 Pittsburgh Institute of Neurodegenerative Diseases, University of Pittsburgh, Pittsburgh, PA, United States of America; 3 Institute of Biotechnology, HiLIFE, University of Helsinki, Helsinki, Finland; Thomas Jefferson University, UNITED STATES

## Abstract

Parkinson’s disease is associated with the loss of dopamine (DA) neurons in ventral mesencephalon. We have previously reported that no single neurotrophic factor we tested protected DA neurons from the dopaminergic toxin 1-methyl-4-phenylpyridinium (MPP^+^) in dissociated cultures isolated from the P0 rat substantia nigra, but that a combination of five neurotrophic factors was protective. We now report that cerebral DA neurotrophic factor (CDNF) and a variant of neurturin (NRTN), N4, were also not protective when provided alone but were protective when added together. In cultures isolated from the substantia nigra, MPP^+^ (10 μM) decreased tyrosine hydroxylase-positive cells to 41.7 ± 5.4% of vehicle control. Although treatment of cultures with 100 ng/ml of either CDNF or N4 individually before and after toxin exposure did not significantly increase survival in MPP^+^-treated cultures, when the two trophic factors were added together at 100 ng/ml each, survival of cells was increased 28.2 ± 6.1% above the effect of MPP^+^ alone. In cultures isolated from the ventral tegmental area, another DA rich area, a higher dose of MPP^+^ (1 mM) was required to produce an EC_50_ in TH-positive cells but, as in the substantia nigra, only the combination of CDNF and N4 (100 ng/ml each) was successful at increasing the survival of these cells compared to MPP^+^ alone (by 22.5 ± 3.5%). These data support previous findings that CDNF and N4 may be of therapeutic value for treatment of PD, but suggest that they may need to be administered together.

## Introduction

Neurotrophic factors (NTFs) have been intensely investigated as possible treatments for various neurological diseases including Parkinson’s disease (PD) [1, 2], Huntington’s disease [3], amyotrophic lateral sclerosis [4, 5], and Alzheimer’s disease [6]. In PD, the dopamine (DA) neurons located in the substantia nigra (SN) are especially vulnerable, and their loss is associated with many of the motor deficits accompanying the disorder [7]. NTFs have been investigated for more than twenty years as protective factors in models of PD. However, although preclinical studies indicate that glial cell line-derived neurotrophic factor (GDNF) and the related factor neurturin (NRTN) can protect DA neurons and restore them after their initial exposure to toxins in models of PD, clinical studies have been disappointing [1, 2, 8]. The failure of these studies may have occurred for a variety of reasons, including a low rate of diffusion of NTFs to surrounding tissue, perhaps due to their binding to heparin sulfate [9–12], an inadequate number of remaining DA neurons in the SN and/or fibers innervating caudate putamen in the advanced stages of the disease in participants in the clinical trial (see reviews [1, 13]), or the clinical trials were too brief to show possible efficacy [14]. However, we postulate an additional reason for the failure of these trials—that multiple NTFs are required to mobilize the cellular machinery needed to protect affected DA neurons from toxic insults, and we have previously reported data that support this explanation [15]. In the present study we examined cerebral DA neurotrophic factor (CDNF) and an engineered form of the NRTN protein, termed N4, using an *in vitro* model of DA loss: dissociated DA neurons obtained from rat pups at postnatal day 0 (P0) and exposed to N-methyl-4-phenylpyridinium iodide (MPP^+^).

CDNF, along with mesencephalic astrocyte-derived neurotrophic factor (MANF), is part of a recently discovered family of NTFs [16] that appear to be at least as potent as the well-studied GDNF against certain toxins [17, 18], although acting via a distinct mechanism [19]. CDNF increases survival in a catecholaminergic cell line, PC12 cells, from cell death induced by either the DA neurotoxin 6-hydroxydopamine (6-OHDA) [20, 21] or methamphetamine [22]. The NTF also protects DA neurons when administered prior to 6-OHDA in both rats [22–25] and nonhuman primates [18] or to MPTP-treated mice [26].

NTRN, along with GDNF, artemin, and persephin, are members of the GDNF family, a subset of the transforming growth factor beta (TGFβ) superfamily of growth factors. NRTN was first identified in 1996 [27] and has many of the properties of GDNF on DA neurons. For example, NRTN increases the survival of DA neurons from SN in culture and protects DA neurons against 6-OHDA and 1-methyl-4-phenyl-1,2,3,6-tetrahydropyridine (MPTP) in rodents and non-human primates [28–31] (see also review by Lindholm and co-workers [32]). However, as in the case of GDNF, despite the promising results with NRTN in models of PD, clinical trials that have used an adeno-virus delivery of the NRTN have not been as successful [33, 34]. In order to increase the bioavailability of NRTN, a new variant has been developed by Mart Saarma’s research group together with Richard Penn and termed N4. This factor retains the NTF properties of NRTN, but has both reduced binding to heparin sulfate or heparin sulfate proteoglycans and improved stability [35, 36].

Based upon our previous observation of a combination of trophic factors protecting DA neurons from MPP^+^ [15], we have now examined CDNF and N4, both separately and together added to cultures isolated from the SN or the ventral tegmental area (VTA) prepared from P0 rat pups. Our findings suggest that whereas neither of these NTFs are effective when added to our culture preparation separately, they are neuroprotective when administered together prior to the addition of MPP^+^. These results may be of clinical significance when designing NTF-based therapies in PD.

## Methods

### Reagents

All reagents were purchased from Sigma/Aldrich (St. Louis, MO, USA) unless otherwise specified.

### Culture preparation

Culturing procedures were carried out in accordance with the NIH Guide to the Care and Use of Animals and approved by the University of Pittsburgh Animal Care and Use Committee. Timed-pregnant Sprague Dawley rats were purchased from Charles River (Wilmington, MA, USA) and housed individually at the University of Pittsburgh in standard microisolater cages with food (Prolab Isopro RMH 3000, LabDiet, St. Louis, MO, USA) and water *ad libitum* under a 12-hour dark/light cycle and allowed to give birth.

Postnatal rat cultures were prepared as previously described [37] with minor modifications. In brief, P0 rat pups were euthanized by decapitation and the brains were isolated under sterile conditions into a cold Gey’s Balanced Salt Solution. Coronal sections of the mesencephalon were then obtained using a scalpel blade. The SN and VTA were isolated separately from these sections under a dissecting microscope with micro-dissection blades, and the tissue digested with papain (Worthington Biochemical, Lakewood, NJ, USA) and mechanical force. The slurry of cells was passed through a concentration gradient to concentrate neurons and remove debris. The live cells were then determined by trypan blue exclusion and 30,000 live cells/well were plated on 16-well Nunc chambered slides (Thermo Fischer Scientific, Pittsburgh, PA, USA) coated with 200 μg/ml poly-d-lysine and 5 μg/ml laminin (Invitrogen, Carlsbad, CA, USA) in feeding media containing 2% rat serum prepared from the dam post-mortem, 2% fetal bovine serum (Atlanta Biologicals, Norcross, GA, USA), GEM 21 NeuroPlex supplement (Gemini Bio-Products, West Sacramento, CA, USA), 0.225% glucose, 1 mM L-glutamine, 100 units/ml penicillin (Life Technologies, Thermo Fischer Scientific, Pittsburgh, PA, USA), 100 μg/ml streptomycin (Life Technologies), 10 mM Hepes, and 0.9 mM sodium pyruvate in basal medium eagle (BME). Cultures were maintained in a 37°C water jacket incubator (Forma Scientific, Inc., Marietta, OH, USA) in an atmosphere containing 5% CO_2_ in equilibrium with H_2_O. Cultures survived for at least 6 days *in vitro* (DIV 6) under basal conditions without significant loss of DA neurons as defined by immunostaining for tyrosine hydroxylase (TH), the rate limiting enzyme in DA synthesis [38]. Thus, to maximize the number of cells in our studies for the MPP^+^ toxicity, we started treatment at DIV4 and determined the number of DA neurons 48 hrs later.

### Exposure of primary cultures to NTFs to determine their effect on basal survival

Recombinant human (rh)CDNF expressed and purified from mammalian CHO cells (10–1000 ng/ml), human neurturin mutant, N4 (10–1000 ng/ml) (gift from NTF Therapeutics, Inc., Chicago, IL, USA), wild type NTRN (10–1000 ng/ml) (Carrier Free, R&D Systems, Minneapolis, MN, USA) or the appropriate vehicles were added to the cultures on the day of preparation (DIV0) within 60 min after plating the cells. The vehicles in these studies were phosphate buffered saline (PBS) (10 mM Na_2_HPO_4_, 1.76 mM KH_2_PO_4_, 26.83 mM KCl, 137 mM NaCl) for CDNF, 10 mM sodium citrate/150 mM NaCl for N4 or 4 mM HCl for NTRN. Cultures were fixed on DIV6 in 4% paraformaldehyde (Electron Microscopy Sciences, Hatfield, PA, USA) and 4% sucrose in PBS) for 30 min, then immunostained for TH, and microtubule-associated protein 2 (MAP2) to assess total neurons, and Hoechst 33258 to assess total cells.

### Exposure of culture to MPP^+^ and NTF

At DIV4, cultures were exposed to MPP^+^, a neurotoxin that enters DA neurons through its high affinity transporter and interferes with complex I of the mitochondrial transport chain [39–41]. The concentrations of MPP^+^ used were those previously shown to be effective [15], 1 or 10 μM for SN cultures and 1 mM MPP^+^ for VTA cultures. We have found that long term exposure to toxins can increase non-specific damage by either MPP^+^ [15] or 6-OHDA [42]. MPP^+^ or sterile water, the vehicle for MPP^+^, were added to duplicate wells 60 min after the media had been removed and replaced with fresh feeding media. After a 30-min incubation, all the media was again removed, the wells washed once with feeding media, fresh feeding media added, and the slides returned to the 37°C incubator.

We examined either a *protection* model or a *restoration* model in our studies. To assess *protection*, NTFs (100–500 ng/ml) or the appropriate vehicle control were added 1 hour *prior* to MPP^+^ exposures and again after removing the MPP^+^ at the same concentrations as used for pretreatment. To assess *restoration*, the NTFs (100–500 ng/ml) were added to the cultures only immediately *after* the removal of MPP^+^. In either case, the cultures were fixed 48 hrs after the MPP^+^ treatment and immunostained for TH, MAP2, and Hoechst.

### Immunostaining

Slides were washed three times in immunocytochemistry (ICC) wash buffer, a solution of 0.1% Tween 20 (BioRad Laboratories, Hercules, CA, USA) and sodium azide in PBS. Next, the slides were incubated for 1 hr in ICC blocking buffer, a solution of 5% BSA (Sigma), 0.1% glycine, 5% goat serum (Jackson ImmunoResearch Laboratories, West Grove, PA, USA), and 0.3% Triton x-100 (BioRad) in PBS with sodium azide. Slides were then incubated in primary antibody overnight. To label the DA neurons we used a rabbit anti-TH antibody (1:5000, Phosphosolutions, cat # 2025-THRAB, Aurora, CO, USA) and to label all neurons we used a chicken anti-MAP2 antibody (1:1000 Fitzgerald Industries International, cat # 20R-2857, Acton, MA, USA). Slides were washed three times in PBS/Tween-20, and then incubated for 2 hr in ICC blocking buffer containing a fluorescently labeled antibody Alexa Fluoro 647 goat anti-chicken (1:1000, Invitrogen, cat # A-21449, Grand Island, NY, USA), Alexa Fluoro 488 goat anti rabbit (1:1000, Invitrogen, cat # A11034), and nuclear stain Hoechst 33258 at 10 μg/ml. All slides were then washed 3 times following antibody treatment with wash buffer and cover slipped with Fluoromount-G (SouthernBiotech, Birmingham, AL, USA).

### H^3^DA uptake

We measured H^3^DA uptake to examine the function of the high affinity DA transporter (DAT) using standard techniques [43]. Specifically, cultures from the SN and VTA were treated on DIV4. Cultures were incubated for 1 hr in media at 37°C containing a vehicle of sodium citrate/PBS, 40 μM nomifensine, an inhibitor of DAT, or the combination of CDNF and N4 (100 mg/ml each). After the 1 hr incubation, the solution was changed to Dulbecco’s PBS (DPBS) (Life Technologies) containing 5 μM glucose and 100 nM H^3^DA at 37°C for 15 min. The cultures were then placed on ice and washed 3 times with cold DPBS with glucose followed by addition of an extraction solution consisting of 33% ethanol and 0.4 N perchloric acid for 15 min at 37°C to release the total tritium. The extraction solution was then collected into scintillation vials filled with ScintiSafe cocktail (Thermo Fisher Scientific). Total tritium present in the solution was then assessed on a scintillation counter (LS6500, Beckman Coulter, Pasadena, CA, USA).

### Data collection and image analysis

Low magnification/high resolution images were taken with MetaMorph Imaging Software (7.4, Universal Imaging, Downingtown, PA, USA) using a Retiga 1300R digital CCD camera (QImaging, Burnaby, British Columbia, Canada) on a Nikon TE 2000 inverted fluorescent microscope (Melville, NY, USA) at various magnifications, and enhanced in Photoshop 6.0 (Adobe System Incorporated, San Jose, CA, USA). These images captured approximately 90% of an individually treated well. Cells were counted using MetaMorph software after verifying the accuracy via manual counting of cells in at least one well for every experiment.

### Statistical analysis

Statistical significance was determined by analysis of variance (ANOVA) followed by the appropriate two-sided post hoc test using SPSS Software (v 25 IBM, New York, NY). Results are presented as mean ± SEM. To assess basal survival of the cells, the ANOVA was done on data normalized to the untreated control in each experiment followed by Dunnett’s post hoc test. For protection analysis, two-way ANOVA for trophic factor and toxin treatment was performed on data normalized to the vehicle control within each experiment. The post hoc analysis was done with the Bonferroni correction for multiple comparisons. The analysis of the uptake data was performed on the raw data using one-way ANOVA followed by Bonferroni correction post hoc test. The numbers of experiments are indicated within the text.

## Results

### Effect of CDNF, NRTN, and N4 on basal survival

Dissociated primary cultures contain a variety of cells both neuronal and glial. We focused on the effects of the trophic factors on the survival of neurons over a total of six days without media changes. Whereas glial cells divide in cultures, thus increasing their numbers, neurons do not divide but slowly die even under the best culture conditions [38]. Thus, comparisons must be made to baselines obtained from cultures incubated for the same amount of time. To label the neurons we used immunofluorescent detection for MAP2, which labels both DA neurons and non-DA neurons, and TH to specifically label the DA neurons.

**Fig 1:**
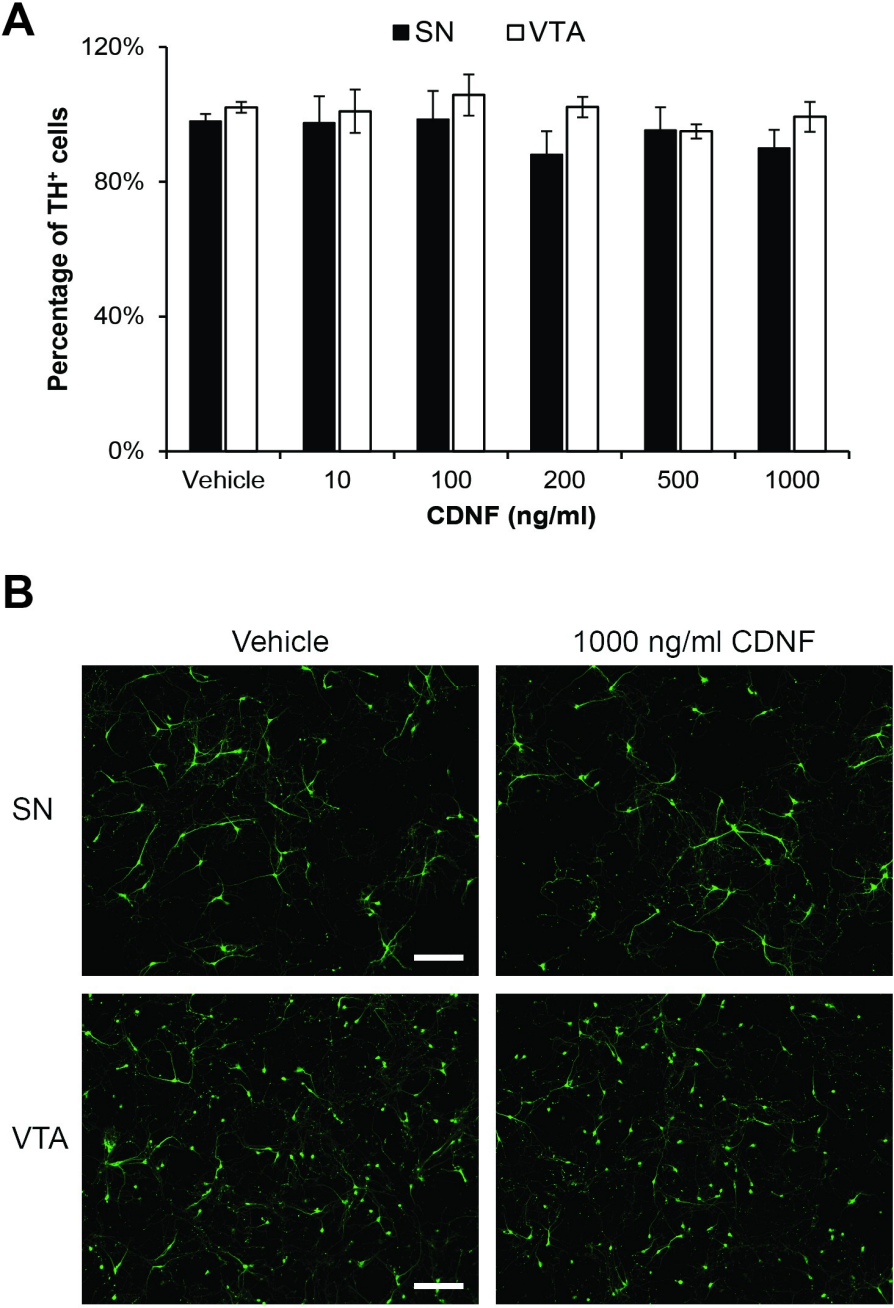
Effect of CDNF on basal survival of DA cells as indicated by TH^+^ immunoreativity cells after six days in culture. A) Neither cells isolated from the SN (closed bars) nor from the VTA (open bars) were affected by CDNF (0–1000 ng/ml) added the day of culture preparation. Graph represents average of cells compared to untreated control; n = 4 experiments per condition. B) Representative images of cells isolated from the SN (top) or VTA (bottom) cultured for 6 days with or without 1000 ng/ml of CDNF immunostained for TH (green). Scale bars indicate 200 microns.

**Fig 2:**
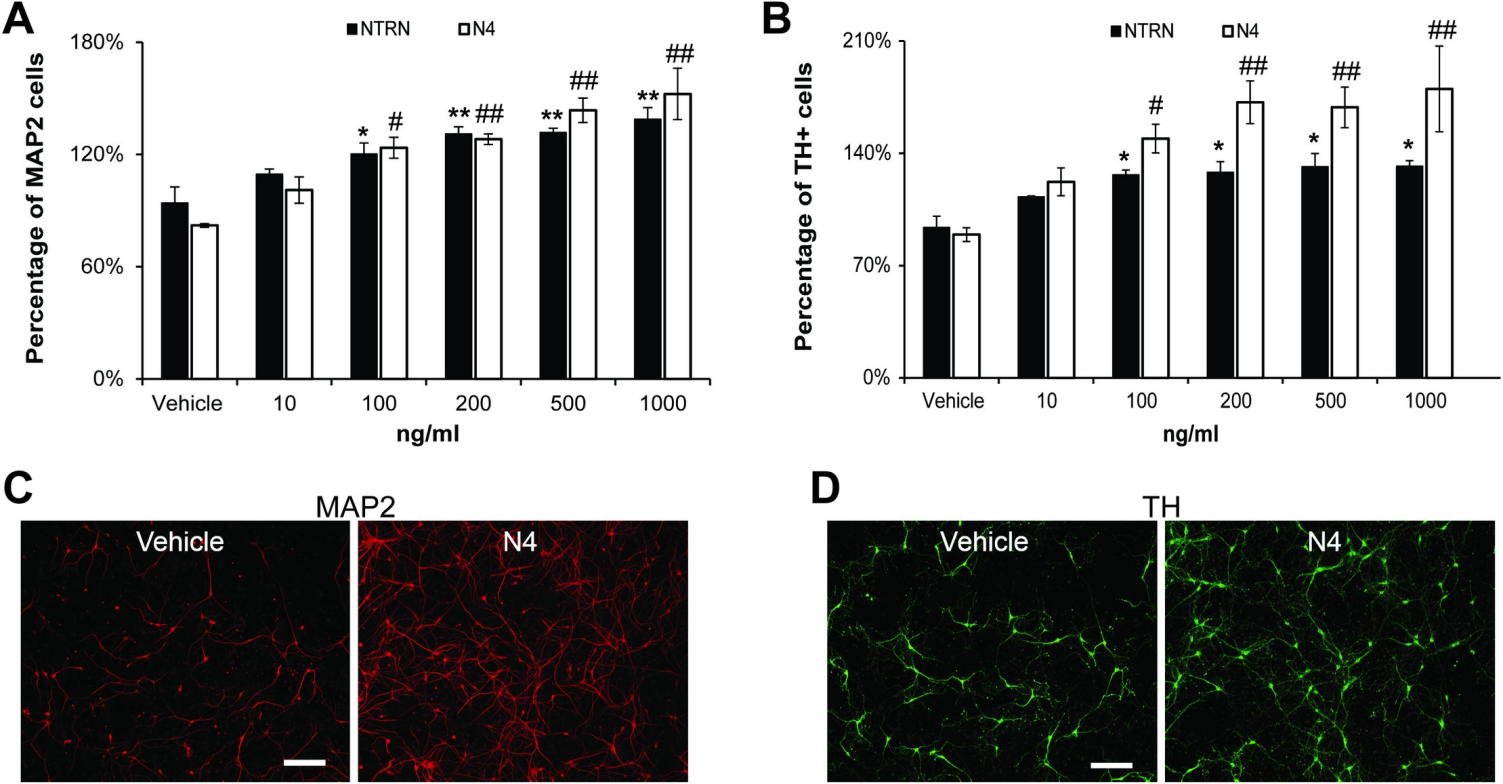
Effect of NRTN and variant N4 on basal survival of all neurons from the SN as indicated by MAP2 (A) or DA neurons as indicated by TH (B). Both NTRN (closed bars) and N4 (open bars) increased the survival of total neurons and TH^+^ neurons isolated from the SN after six days in culture. The NTFs were added the day of culture preparation within an hour of culture preparation.(* equals p<0.05 or ** = p<0.005, # < 0.05 or ## < 0.005 ANOVA followed by Dunnett post hoc test on data normalized to untreated control within in each treatment; n = 3 to 4 experiments per condition.) Representative images of cells isolated from the SN cultured for 6 days with or without 1000 ng/ml of N4 immunostained for MAP2 (C, red) and TH (D, green). Scale bars indicate 200 microns.

CDNF (10–1000 ng/ml) alone had no effect on the survival of either the DA neurons or the total number of neurons present in the cultures from either the SN or VTA (Fig 1A), nor was there any noticeable impact on neuronal morphology (Fig 1B). Likewise, neither N4 nor NTRN significantly increased the number of either DA neurons or total neurons isolated from the VTA under basal conditions when added separately. In contrast, 100 ng/ml NTRN significantly increased the survival of MAP2^+^ neurons in SN cultures under basal conditions by 20.3 ± 5.8% compared to untreated cultures (p = 0.02, n = 3, Fig 2A) with a maximal increase in survival of 38.8% at a NTRN concentration of 1000 ng/ml (p < 0.001, n = 3, Fig 2A). NTRN also increased DA neuron survival by 26.6 ± 5.3% (p = 0.036, n = 3, Fig 2B) but, as with the MAP2^+^ cells, this effect plateaued at doses of 100 to 1000 ng/ml to 31.9 ± 5.4% (Fig 2B). N4 increased the survival of MAP2^+^ cells by 23.6 ± 5.6% at 100 ng/ml (p = 0.015, n = 4) and up to 52.4 ± 13.7% with 1000 ng/ml compared to the no treatment control (p<0.0001, n = 4, Fig 2A and 2C). The survival of DA neurons in the SN was increased by even greater percentages with N4, 49.2 ± 8.9% and 80.2 ± 26.7%, for 100 ng/ml (p = 0.03, n = 4) and 1000 ng/ml (p<0.001, n = 4), respectively (Fig 2B and 2D).

### Effect of CDNF and N4 on MPP^+^ toxicity in SN cultures

**Fig 3:**
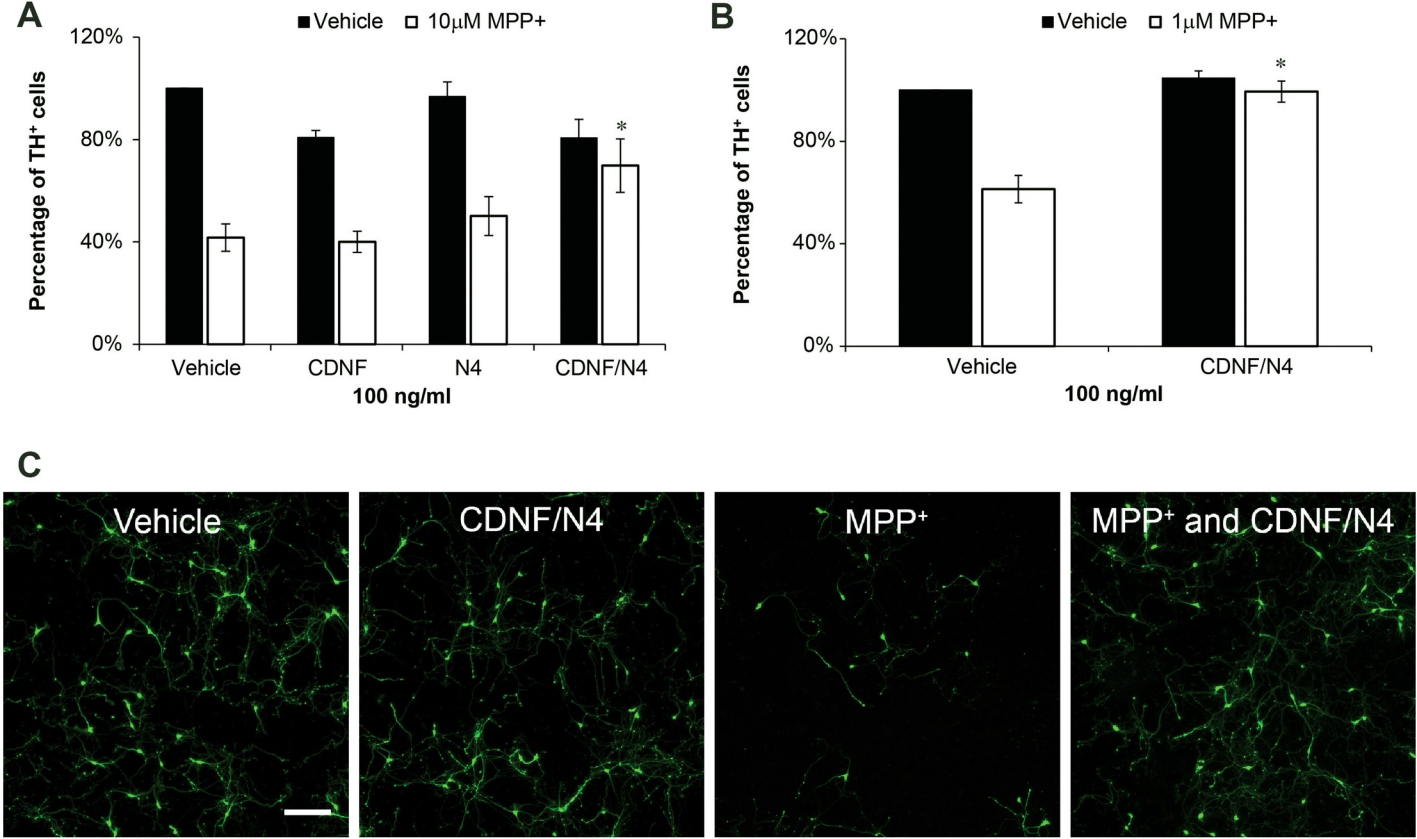
Protection of DA neurons as indicated by TH isolated from the SN from MPP^+^. 100 ng/ml of CDNF and N4 present 1hr before and for 48 hr after MPP^+^ exposure had a protective effect of the survival of DA neurons. (A) Neither CDNF nor N4 individually had a significant effect on TH^+^ cells survival. However, when the two NTFs were added together the number of TH^+^ cells increased by 28.2 ± 10.5% against 10 μM MPP. (B) The combination of CDNF and N4 completely protected DA neurons against 1 μM MPP^+^ (* = p<0.05 two-way ANOVA followed by post hoc test with Bonferroni correction on data normalized to the vehicle control, n = 3 or 4 experiments per condition). (C) Representative images of immunostaining of DA neurons for TH (green) in vehicle treated and CDNF/N4 (100 ng/ml of each) in the presence or absence of 10μM MPP^+^. The scale bar indicates 200 microns.

In our previous studies we observed that concentrations of 10 μM MPP^+^ or lower resulted in a selective decrease in DA neurons in SN cultures after 48 hrs [15]. In order to assess neuroprotection in this paradigm, NTFs were added 1 hr *prior* to MPP^+^ (1.0–10 μM) exposure and remained present until the cultures were fixed and immunostained 48 hrs later. This paradigm was different from that used in our study of basal survival in which the trophic factor was added the day the cultures were prepared and remained until the cultures were fixed for a total of 6 days without any media change or refreshing of trophic factors. The exposure to MPP^+^ resulted in a 60 ± 5.4% loss of DA neurons for 10 μM. At a concentration of 100 ng/ml neither CDNF nor N4 had a significant effect on the number of DA neurons when added individually in the toxicity paradigm. On the other hand, when 100 ng/ml CDNF and N4 were added simultaneously, we observed a 28.2 ± 10.5% (p = 0.04, n = 4) increase in the survival of the toxin-treated DA neurons (Fig 3A and 3C). When SN cultures were treated with a lower concentration of MPP^+^ (1 μM), a 38.7 ± 3.1% DA loss was reversed with 100 ng/ml of CDNF and N4 added together (Fig 3B).

### Effect of CDNF and N4 on MPP^+^ toxicity in VTA cultures

**Fig 4:**
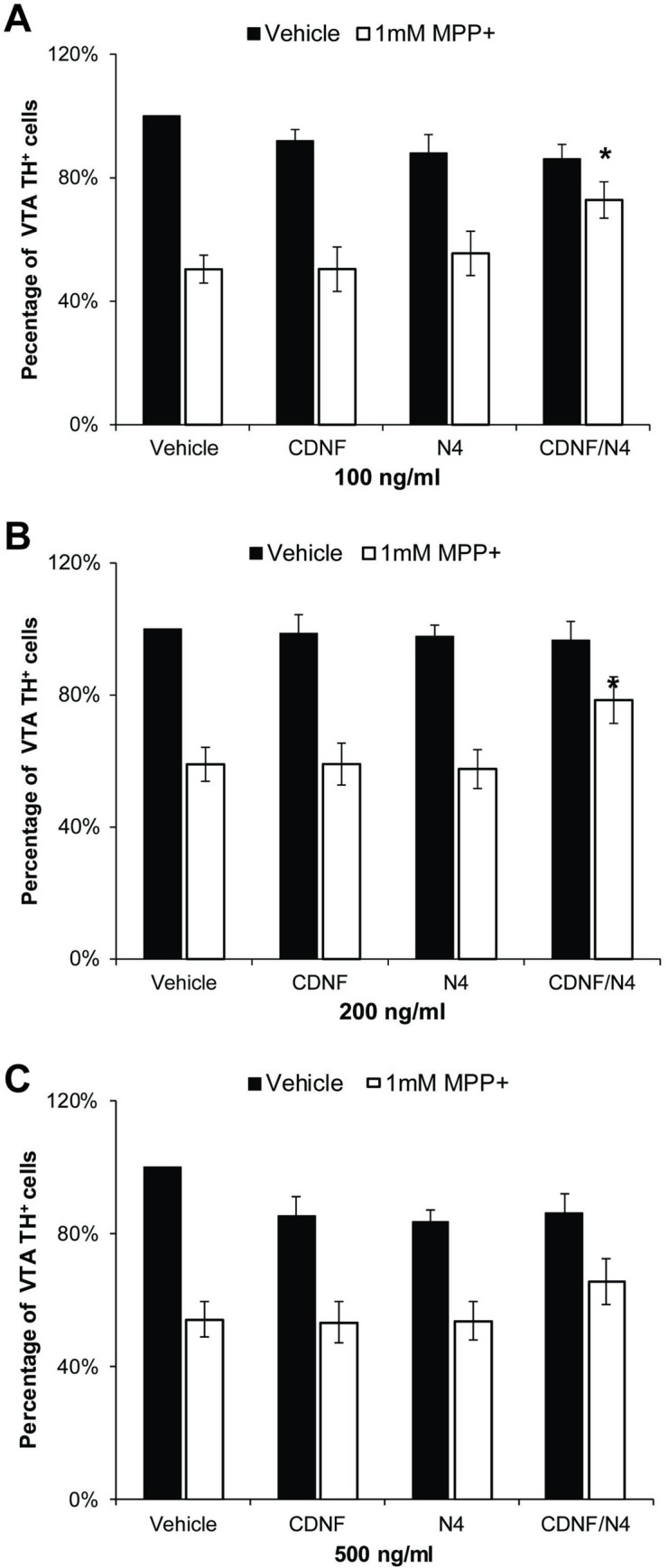
Protection by the combination of CDNF and N4 of DA neurons and indicated by TH isolated from the VTA. NTFs were added 1 hr prior to the addition of 1 mM MPP^+^ and for 48 hrs thereafter at concentrations of (A) 100 ng/ml and (B) 200 ng/ml in cultures isolated from the VTA. The combination of CDNF and N4 significantly increased survival of DA neurons (*p < 0.05, two-way ANOVA followed by post hoc test with Bonferroni correction on the cell count, n = 6–7 experiments). (C) 500ng/ml of CDNF and/or N4 did not increase survival but did cause some but not significant basal cell loss.

We have found that in cultures isolated from the VTA, a higher concentration of MPP^+^ is required to kill DA neurons than in SN neurons [15]. 1 mM MPP^+^ significantly decreased the number of DA neurons by 49.3 ± 4.5% (p = 0.006, n = 6). As in the SN cultures, CDNF and N4 alone at concentrations of either 100 or 200 ng/ml did not increase the survival of in the VTA DA neurons (Fig 4A and 4B). In contrast, the combination of CDNF and N4 at either 100 or 200 ng/ml increased survival by 22.5 ± 5.9% and 19.7 ± 6.3%, respectively (p < 0.05, n = 6 or 7) (Fig 4A and 4B). Although both 100 and 200 ng/ml concentrations were protective, the higher concentration did not offer additional protection. Surprisingly, treatment with 500 ng/ml also did not have any further effect on cell survival and, indeed, some cell death was observed in the control NTFs alone treated cultures, although it was not statistically significant (Fig 4C).

We also did extensive experiments using a paradigm of *restoration*, in which the trophic factors were added individually and in combination only *after* the removal of the MPP^+^ in both the SN and VTA cultures. Although we observed trends in the increase number of DA cells, we did not find a consistent significant restoration.

### H^3^DA uptake

**Fig 5:**
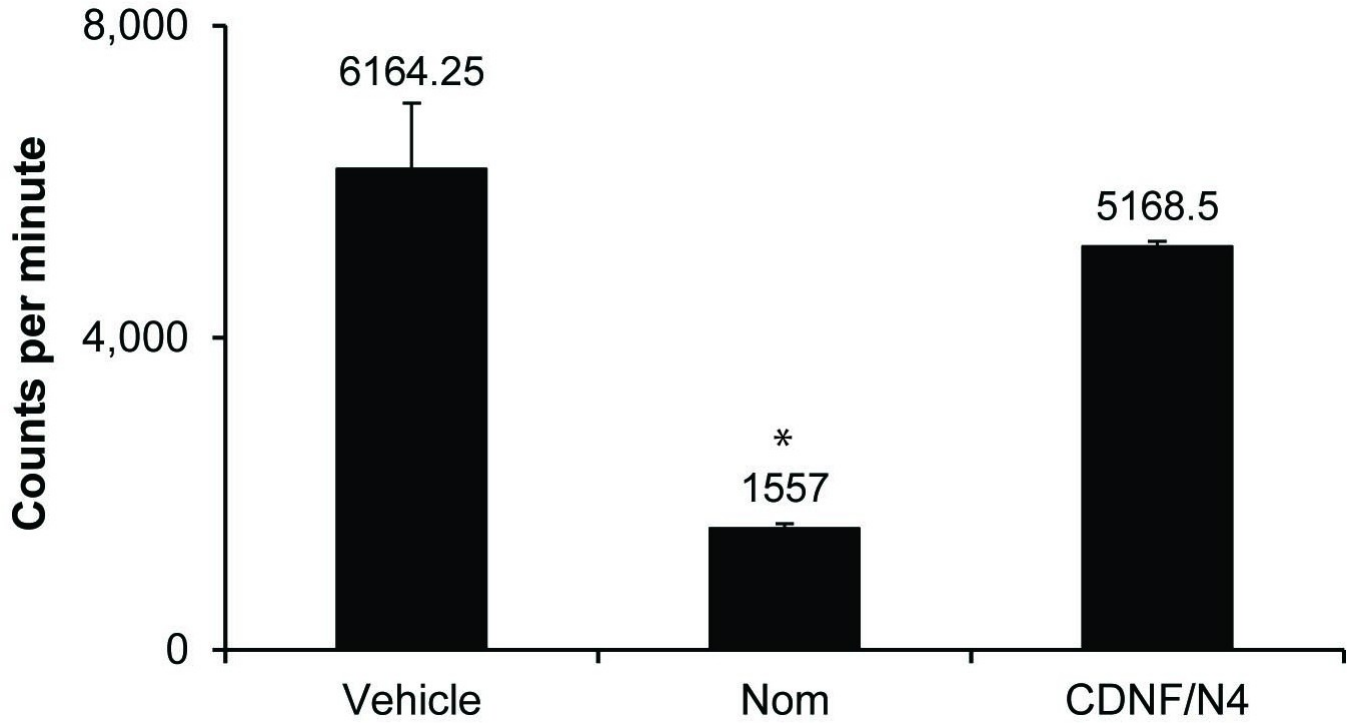
The function of the high affinity DA transporter (DAT) in the presence of 40 μM nomifensine or the combination of 100ng/ml CDNF and N4. A 1hr incubation of nomifensine significantly decrease uptake of H^3^DA in cultures isolated from the SN. (* = p < .01 Bonferroni post hoc analysis, n = 2). The trophic factor combination had no effect on H^3^DA uptake indicating that protection was not a result of inhibition of DAT.

One possible explanation for apparent NTF-induced protection from MPP^+^ is an inhibition of the toxin’s entry into the cells, which occurs primarily via DAT [44]. To examine this possibility, we measured the uptake of H^3^DA after a 1 hr incubation with the NTF combination of CDNF plus N4 or vehicle and compared it to cells incubated with 40 μM nomifensine. Total tritium uptake into the cells was taken as a measure of H^3^DA uptake. Nomifensine decreased tritium uptake by 74.1 ± 0.05% (p<0.01, n = 2), whereas CDNF plus N4 did not significantly change tritium uptake in either the SN or VTA cultures (Fig 5).

## Discussion

### Effects of CDNF and N4 on primary DA neurons under basal conditions

PD is typically associated with the degeneration of DA neurons in the ventral mesencephalon. Within that structure, the DA neurons of the SN usually die first; then, as the disease progresses, the neurons in the VTA also die [45]. We had two main objectives when we began to examine CDNF and the NRTN mutant, N4, in postnatal primary cultures. The first was to characterize the effect of CDNF and N4 under basal conditions as well as to compare N4 to NTRN. There have already been several published reports that have employed *in vitro* models to examine the effects of CDNF [20, 21, 46]. For example, CDNF has been shown not to effect basal survival of motor, sensory, or sympathetic neurons ([23] see supplement). However, studies of *embryonic* DA neurons generally do not examine SN and VTA separately. In contrast, in the studies reported here we employed *postnatal* cultures from the ventral mesencephalon, which allowed us to examine differentiated neurons and, further, to separate the SN and VTA. This separation is critical as we have extensively examined SN versus VTA in regard to basal survival in culture and in the presence of toxins [15, 37, 38, 42, 47]. Moreover, in an adult rat the VTA has 1.5 times the number of DA neurons than the SN [48], and thus a culture prepared from the ventral mesencephalon is also likely to have a higher percentage of VTA DA neurons. In addition, we have found that, during the process of preparing cultures from the SN and VTA, separately, fewer DA neurons survive in the SN than in the VTA, further diluting the population of TH^+^ SN neurons. The differences in the SN and VTA are not limited to the DA neuronal population. Glial cells which include astrocytes, microglial, and oligodendricytes have different ratios in the two regions (67).

Using separate primary cultures of SN and VTA, we found that CDNF did not have any effect on the survival or morphology of either DA or non-DA neurons from either region under basal conditions. This indicates that CDNF does not have any impact on differentiation, maturation, or morphology of DA neurons *in vitro*, and corresponds to the observation that CDNF did not increase neurite complexity or formation of varicosities [18, 23, 24]. Should CDNF be used as an intervention for PD, these characteristics might be advantageous because there is always the possibility that adding exogenous trophic factors could lead to innervations of other regions of the brain (see our previous discussion of this point [49]) or tumorigenesis [50, 51].

In contrast to CDNF, N4 acted similarly to NRTN [52] and GDNF [37] in that it increased the survival of DA neurons in the SN under basal conditions, although no effect of N4 was detected on DA neurons in the VTA under these conditions. The increased survival of DA neurons in the SN was comparable to the magnitude of that we had previously observed with GDNF at a similar concentration [37] and higher than our present observations of NTRN in the same culture system. This is consistent with the fact that GDNF and NRTN are both members of the TGFβ superfamily [27, 36, 52, 53]. However, in contrast to the reported lack of effect of GDNF on non-DA midbrain neurons, both NTRN and N4 also increased the survival of non-DA neurons, an effect that was only observed in the SN cultures. The increase in the number of DA neurons, being approximately 32% of neurons in the cultures, was not large enough to account for the NTRN- or N4-induced rise in total of MAP2^+^, indicating that neurons other than DA neurons were also affected. As noted above, most published culture preparations do not separate the SN and VTA [28, 36, 54] and we are not aware of any previous reports of effects of NRTN itself on non-DA neurons from the SN or VTA when those areas were examined separately. Although we did not attempt to identify the type of the non-DA neurons affected by N4, NRTN has been shown to increase survival of cholinergic neurons in embryonic basal forebrain cultures [55], and cholinergic neurons are also found in the SN [56].

### The effects of CDNF and N4 in the presence of MPP^+^

The second objective of our studies was to determine if CDNF or N4 could protect against MPP^+^-induced toxicity. In our studies, we exposed cultures to MPP^+^ for only 30 min, rather than leaving the toxin in the medium for 24 hrs or more as is often done by others, because we had found that leaving MPP^+^ for extended time resulted in non-specific cell death [15]. Using this paradigm, we did not observe any protection of DA neurons against MPP^+^ by either CDNF or N4 applied individually at concentrations of 100 or 200 ng/ml in the cultures of cells isolated from the SN or at concentration of 100–500 ng/ml in the cultures from the VTA [15]. Protection against MPP^+^ was only observed when CDNF and N4 were added together. Moreover, this effect appeared to be synergistic, because it was greater than the total of the small, insignificant protection of adding the individual NTF. We also found a synergistic effect in the cultures prepared from the VTA. Protection of DA neurons from the VTA in cultures that did not include SN has not been previously reported. Although we also examined a restoration model by adding the combination of CDNF and N4 after MPP^+^, the trends in protection the outcomes were not statistically significant.

Our results are in contrast to the ability of CDNF alone to protect against DA-specific toxins such as 6-OHDA [18, 23–25, 57, 58] and MPTP [26, 59] *in vivo*. Similarly, NRTN and N4 have been shown to be protective in *in vivo* models using either 6-OHDA and MPTP [29, 36, 52, 60–64]. This apparent distinction between *in vivo* and *in vitro* results is reminiscent of our own observations with several other NTFs [15]. For example, whereas we were able to confirm the neuroprotective properties of GDNF against 6-OHDA and MPTP *in viv*o [37, 42, 65, 66], in our hands GDNF alone did not protect primary DA neurons from rat ventral mesencephalon when the neurons were exposed to MPP^+^ *in vitro*. Instead, GDNF required the presence of several other NTFs to provide its neuroprotective properties under those conditions.

We have postulated that a critical difference between *in vitro* and *in vivo* models is that *in vivo* a large number of endogenous trophic factors are already present and thus able to interact with the exogenous trophic factors as well as the presence of other cell types, including microglial and astrocytes that have the receptors for various trophic factors [15]. The effect of multiple trophic factors on the survival of DA neurons has only been investigated in relatively few publications to date. In addition to our studies, combinations of TGFβ, SHH, and FGF8 [67]; GDNF and BDNF [68]; GDNF and TGFβ [69–71]; GDNF and VEGF [72]; GDNF and CDNF [19]; and CDNF and MANF [17] have been examined. If our hypothesis is correct it may have important clinical implications. Specific NTF levels are reduced in the brains of patients of several neurological diseases, including PD [73–76], Alzheimer’s [59], schizophrenia [58], and depression [60, 61], and this may preclude the normal interaction between an exogenous NTF and those already present endogenously.

If so, this would suggest that the use of multiple trophic factors, including those that have been genetically engineered to increase their efficacy, such as N4, may be essential for therapeutic treatment of PD. The need for the use of multiple agents to effectively treat a disease would not be unique. Indeed, it has proved essential for the treatment of many forms of cancer [77, 78], HIV/AIDS [79], and pneumonia [80].

## Supporting information

S1 File (XLSX)

S2 File (XLSX)

S3 File (XLSX)
